# Radial Approach Expertise and Clinical Outcomes of Percutanous Coronary Interventions Performed Using Femoral Approach

**DOI:** 10.3390/jcm8091484

**Published:** 2019-09-18

**Authors:** Tomasz Tokarek, Artur Dziewierz, Krzysztof Plens, Tomasz Rakowski, Michał Zabojszcz, Dariusz Dudek, Zbigniew Siudak

**Affiliations:** 1 2nd Department of Cardiology and Cardiovascular Interventions, University Hospital, 17 Kopernika St., 31-501 Krakow, Poland; tomek.tokarek@gmail.com (T.T.);; 2 2nd Department of Cardiology, Institute of Cardiology, Jagiellonian University Medical College, 31-501 Krakow, Poland; 3 Krakow Cardiovascular Research Institute, 30-055 Krakow, Poland; 4 Faculty of Medicine and Health Science, Jan Kochanowski University, 25-317 Kielce, Poland

**Keywords:** experience, registry, all-comers, radial, femoral

## Abstract

We sought to evaluate the impact of experience and proficiency with radial approach (RA) on clinical outcomes of percutaneous coronary interventions (PCI) performed via femoral approach (FA) in the “real-world” national registry. A total of 539 invasive cardiologists performing PCIs in 151 invasive cardiology centers in Poland between 2014 and 2017 were included. Proficiency threshold was set at >300 PCIs during four consecutive years per individual operator. The majority of operators performed >75% of all PCIs via RA (449 (65.4%)), 143 (20.8%) in 50–75% of cases, 62 (9.0%) in 25–50% and only 33 (4.8%) invasive cardiologists were using RA in <25% of all PCIs. Operators with the highest proficiency in RA were associated with increased risk of periprocedural death, stroke and bleeding complications at access site during angiography via FA. Similarly, higher prevalence of periprocedural mortality during PCI with FA was observed in most experienced radial operators as compared to other groups. The detrimental effect of FA utilization by the most experienced radial operators was observed in both stable angina and acute coronary syndromes. Higher experience and utilization of RA might be linked to worse outcomes of PCIs performed via femoral artery in both stable and acute settings.

## 1. Introduction

Radial approach (RA) for percutaneous coronary intervention (PCI) is associated with reduced mortality and access site complications [1,2,3,4,5,6,7,8]. Mounting evidence favoring radial over femoral approach was reflected in the current European Society of Cardiology (ESC) guidelines [9]. The routine use of the RA should be strongly considered, keeping in mind the learning curve associated with the technique [9]. However, wide promotion of RA may interfere with the equally important goal of maintaining expertise and proficiency in the femoral approach (FA), which is essential in a variety of procedures as well as when RA fails. Recently, concerns have been expressed regarding safety and efficacy of FA for interventionist practicing mostly with radial artery [10]. Furthermore, some studies attempted to explain the superiority of PCI with RA by taking into account the operator’s experience and reduced proficiency in FA rather than the radial route per se [10]. Despite the lack of definite evidence for such a phenomenon, the so-called Campeau radial paradox emphasized the role of the operator’s experience as a key determinant of outcomes [10]. However, there are still limited data on clinical outcomes of FA utilization by operators with different procedural volume and dexterity level of RA in an all-comers population. Thus, we sought to evaluate the effect of experience and proficiency with RA on clinical outcomes on PCIs via FA in “real-world” patients with stable angina (SA) and acute coronary syndrome (ACS) enrolled in the Polish National PCI Registry (ORPKI).

## 2. Materials and Methods

A detailed description of the ORPKI national PCI registry was presented previously [7,11,12,13,14]. Briefly, the ORPKI is an electronic database on all percutaneous procedures in interventional cardiology centers performed in Poland and is currently operated by the Jagiellonian University Medical College in Krakow. Clinical and procedural data were prospectively collected from January 2014 to December 2017. Following guidelines, a proficiency threshold has been set at >300 PCIs during four consecutive years per individual operator [9,15]. A total of 539 invasive cardiologists from 151 invasive cardiology centers in Poland were included in the analysis. Procedures performed on patients with cardiogenic shock on admission were excluded from the analysis. Radial and total operator volumes were determined using unique operator’s identification numbers in the ORPKI database. Total volume was calculated for each operator separately as the overall number of PCIs performed during enrollment. The percentage of the use of the RA during PCI was calculated by dividing those two values. Operators were categorized to quartiles according to the percentage of RA utilization during all PCIs. Procedures were performed using either RA or FA depending on the operator’s discretion. Vascular access site for the procedure was defined as the site of successful vascular entry. Procedures involving unidentified or switched access sites were excluded. All procedures were carried out according to local standards of PCI and ESC guidelines wherever applicable. All complications that occurred during procedures were documented prospectively. Periprocedural mortality was defined as the death of any cause during procedure (coronary angiography or PCI). Bleeding complications were defined homogeneously in all centers as any overt, actionable sign of hemorrhage (e.g., more bleeding than would be expected for a clinical circumstance, including bleeding found by imaging alone) that does not fit the criteria for type 3, 4 or 5, but does meet at least one of the following criteria: (1) requiring nonsurgical, medical intervention by a healthcare professional, (2) leading to hospitalization or increased level of care or (3) prompting evaluation [16]. Diagnosis of stroke was established by local physicians. Complete data on stroke type and neurological outcomes were not collected. Adverse events were diagnosed at the operator’s discretion according to definitions and current ESC guidelines [9]. No further evaluation or follow-up was performed after hospital discharge. The analysis was performed for both SA and ACS. All patients provided informed consent for the procedure. The study complied with ethical principles for clinical research based on the Declaration of Helsinki with later amendments. No funding was used to support this registry. 

## 3. Statistical Methods

Continuous variables were expressed using the mean and standard deviation. Normality was assessed by the Shapiro-Wilk test. Equality of variances was assessed using the Levene’s test. Differences between groups were compared using the standard ANOVA (analysis of variance, differences in mean) or the Welch’s ANOVA depending on the equality of variances for normally distributed variables. The Kruskal-Wallis test was used for comparison of non-normally distributed variables. For paired data samples, where the measurement was performed on an interval or a ratio scale the paired Student’s *t*-test was used if differences between pairs were normally distributed, the Wilcoxon signed-rank was used otherwise. Two-sided *p*-values < 0.05 were considered statistically significant. All statistical analyses were performed with JMP^®^, Version 14.2.0 (SAS Institute INC., Cary, NC, USA). 

## 4. Results

**Figure 1:**
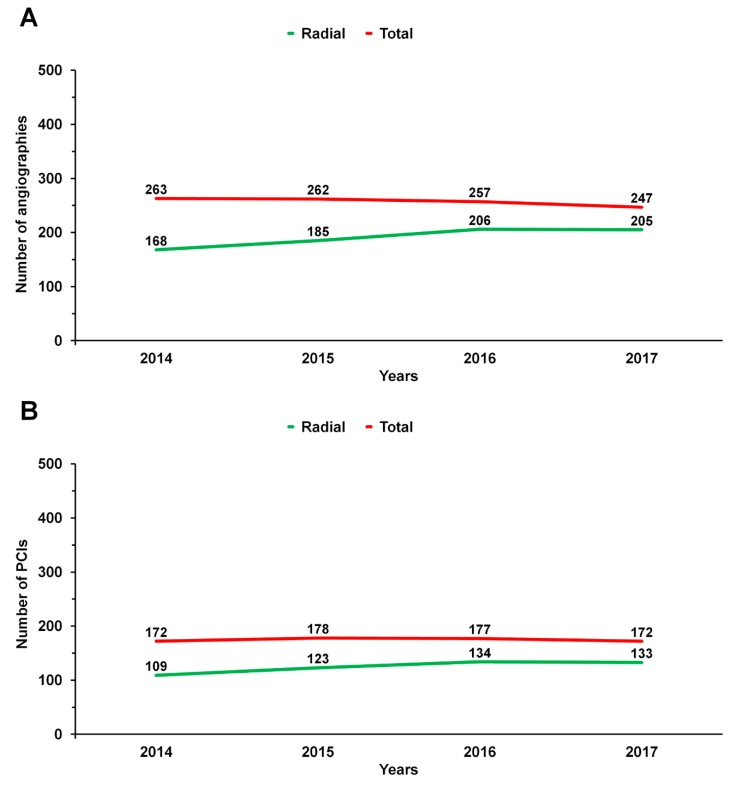
Median operator volume from 2014 to 2017: (**A**) for angiography; (**B**) for percutaneous coronary interventions.

A total of 613,125 angiographies (78.2%) and 311,342 PCI (73.8%) were performed with RA, including 70.4% procedures in SA and 62.5% in ACS. Median operator volume from 2014 to 2017 is presented in Figure 1.

The majority of the operators performed >75% of procedures via RA (angiography and PCI, respectively, 449 (65.3%) and 326 (60.5%) operators). A default femoral operators were the lowest quartile (angiography and PCI, respectively, 33 (4.8%) and 34 (6.3%) operators). Complete baseline clinical and angiographic characteristics of the included patients with treatment administered during PCI are presented in Table 1 and Table 2.

Operators with the highest proficiency and utilization of RA were associated with increased risk of periprocedural death, stroke and bleeding complications at access site during angiography performed with femoral artery. Similarly, the higher periprocedural mortality during PCI with FA was observed in most experienced radial interventionists as compared to other groups. In addition, a trend towards the higher rate of bleeding at the puncture site during PCI procedures was reported in this group (Table 3 and Table 4).

Similarly, a detrimental effect of FA utilization by most experienced radial operators was observed in both SA and ACS settings. However, a trend was observed for the higher periprocedural mortality during PCI in SA and angiography in ACS as well as bleeding complications during PCI for both settings (Table 5 and Table 6). 

Incidence of periprocedural complications in comparison of RA with FA among each quartile of operators is presented in Table 7, Table 8, Table 9 and Table 10. 

## 5. Discussion

The results of our analysis suggest that operators with the highest radial approach volume percentage were more likely to experience periprocedural complications during FA utilization as compared to other operators. The observed results might be related to jeopardizing proficiency in FA in favor of RA adaptation in a daily practice. To the best of our knowledge, this study is, to date, the largest multicenter report describing the impact of change in access site practice towards RA on clinical outcome for PCI undertaken through FA in an unselected cohort of patients. Safety and benefits of RA adaptation in PCI have been previously confirmed [1,2,3,4,5,6,7,8]. However, the change in access site practice from FA to a predominantly RA outlined an impact of experience as key variable influencing outcomes. A relationship between operator/center radial volume and outcomes in PCI has been previously reported [2,5]. Critical appraisals of MATRIX and RIVAL studies suggested that the difference in outcome between FA and RA are at least partially driven by greater adverse events in the radial expert group performing the procedure via FA rather than benefits of the radial route per se [2,5,17]. However, most of the previous studies compared only access types and did not consider the impact of individual operator level [7,18]. A recent large retrospective analysis demonstrated that outcomes were similar between centers transitioned towards RA adaptation and those that remained mainly femoral. After adjustment for case mix, no differences were observed in 30-day mortality and vascular complication rates after PCI with the RA in centers transitioned to RA as compared with those remaining predominantly femoral. However, data were based on retrospective analysis and did not include radial experience at an operator level [17,18]. In contrast, a higher rate of vascular complications (12.5%) was observed in procedures performed with the FA by default radial operators [19]. In another study, the risk of bleeding complications related to access site in patients treated with FA increased after the adoption of RA [18]. Interestingly, the risk increased more in a group with higher adoption of RA as compared to those performing procedures via radial artery less frequently. This might suggest that centers and operators utilizing predominately RA might lose proficiency in performing FA [20]. Another analysis compared access site-related outcomes between a historical group treated only with FA and a contemporary cohort [10]. Patients with FA in the contemporary group experienced more vascular complications compared with patients undergoing PCI with FA in the past (unadjusted rates: 4.68% versus 2.89%; *p* = 0.001; adjusted rates: 4.19% versus 1.98%; adjusted Odds Ratio (OR), 2.16; 95% CI, 1.67–2.81; *p* = 0.001) [10]. This finding was consistent for both diagnostic and therapeutic catheterizations [10]. However, the analysis was limited by the absence of pharmacological data and rate of concomitant femoral vein puncture and sheath size in the historical cohort. Patients selected for FA had more comorbidities than the RA group, thus, risk for major vascular complications in the femoral group was higher. It appears that the risk-treatment paradox was responsible for outcomes rather than a “radial paradox” [21]. Finally, the biggest meta-analysis to date, including 15,615 patients, reported outcomes of PCI via FA in the group of radial expert (RE) and non-radial expert (NRE) [22]. The mortality rate for radial experts was more than double as compared to those less familiar with RA. In a pairwise meta-analysis, the group with high expertise in RA was associated with increased risk of mortality (OR: 1.72, 95% CI: 1.13–2.62; *p* = 0.011) as compared to FA-NRE. In subgroup analysis, FA-RE was linked with increased risk of death (RR: 1.70, 95% CI: 1.24–2.34; *p* = 0.001) as compared to RA, but RA-NRE was not. Similarly, in mixed comparison models, FA-RE was associated with increased mortality compared to other groups. However, in FA-NRE risk of death was not higher as compared to RA-RE and RA-NRE. Furthermore, the risk of major bleeding with FA-RE was the highest among all groups, which could partially explain increased mortality rate. However, these results were limited by the methodology of each included study. There were differences in definitions of radial expertise as well as follow-up intervals [22]. Our analysis is consistent with these findings. Operators were trained to master the RA, but the adaptation of the new access might be attenuated by a loss of femoral proficiency. Higher mortality in PCI via FA in the group of most experienced radial operators might be partially explained by increased bleeding and access-site complications. Discontinuation of antiplatelet or antithrombotic therapy with the potential need for blood transfusions is associated with higher mortality [23]. Nevertheless, a direct cause-effect correlation between periprocedural mortality and bleeding complications cannot be corroborated in our analysis. On the other hand, interesting results were noticed in procedures performed with RA. Higher periprocedural mortality undertaken radially by preferable radial operators might be a marker of low-experienced interventionist not familiar enough with FA. There is also potential bias related to the higher number of operators and procedures performed by radial experts as compared to other groups. This result was observed especially in the ACS setting. Those patients were initially at higher risk of complications. In our study radial experts using FA were associated with a higher rate of periprocedural stroke as compared to other groups. Most evident differences were observed in ACS, as it is a life-threatening condition requiring rapid decisions and treatment. However, no difference in stroke rate was observed in RA procedures despite the level of radial route proficiency. In contrast, a recent study reported a higher rate of stroke during PCI in acute myocardial infarction performed by the operators with less experience with the RA [12]. The percent of PCIs using the radial artery per operator (OR: 0.981 per 1% increase, 95% CI: 0.967–0.997; *p* = 0.02) was an independent predictor of periprocedural stroke. In opposite to data from the ACCOAST trial, there was no impact of the RA per se on the risk of stroke [12,24]. Furthermore, in a recent meta-analysis, RA was not associated with an increase in neurological complications as compared to FA [25]. Another large study even suggested a lower occurrence of periprocedural stroke in RA as compared to procedures via FA [26]. Operator volume is often used as a surrogate for quality measurement, but there is a discrepancy in definitions of high- or low-volume operators. Data derived from centers in North America has determined that the threshold is a minimum of 50 RA procedures per year to maintain competency in PCI [27]. This would be considered a low volume in countries with RA as a default access site. The British Cardiovascular Interventional Society advocates 150 procedures over two years to maintain competency [28], while according to ESC guidelines, an independent operator should perform >75 PCIs annually at high-volume centers (>400 PCIs) with on-site cardiac surgery [9,15]. Thus, conflicting results might be obtained regarding the effect of FA utilization by operators with diverse RA proficiency. The emphasis of the impact of the operator’s radial volume should be interpreted with caution, as the volume is not a surrogate for quality and merely one of the variables influencing outcome. RA may be preferentially selected by younger operators while still on training. Older and most advanced operators might be mainly assigned to more difficult procedures in high-risk individuals requiring FA utilization. Thus, this group could be affected with an increased risk of complications. In addition, a low number of procedures per year in this group of operators will not adequately describe experience and proficiency in PCI. Lifetime learning and years of experience might play an important role in a decreased number of adverse events [29]. Finally, techniques for PCI have become much more standardized and dedicated tools facilitate complex PCI procedures. Thus, outcomes might become less influenced by the skills of the individual operator and more dependent on the advances in technology. Despite overwhelming data supporting the superiority of RA over FA, caution should be paid to maintain FA proficiency. Attenuated experience in PCI with FA could be a potential problem in the nearest future. Presented results should not prompt to reverse the adoption of the RA. However, this issue certainly requires further investigation. The identification of a minimum volume for optimal clinical outcome should be elaborated for operators on training to incorporate an adequate proportion of both RA and FA in their practice. Low-risk patients and procedures requiring FA should provide a volume of cases to maintain sufficient training in femoral artery puncture. Furthermore, precise guidelines and recommendations are needed. It will allow training courses and certification organizations to define standards for proficiency and expertise.

## 6. Limitations

Some important limitations should be addressed. The main is the non-randomized design with all the inherent bias. The possibility of unmeasured confounders affecting the outcome cannot be excluded. Our study is hypothesis generating. Thus, a causal relationship between an operator’s radial experience and the outcome of treatment cannot be definitely made. Furthermore, there is potential bias resulting from lifetime experience in percutaneous procedures. Impact of the paucity of in-hospital data on clinical outcomes cannot be ruled out. There is a lack of angiographic data describing lesion type and morphology as well as devices specifications. Size of vascular sheaths used during PCI and utilization of closure devices were not reported. Furthermore, data after discharge from the hospital were not captured in this registry. The operator volume might also be underestimated by performing PCI in other hospitals not included in the national database (e.g., abroad). Finally, there are some inconsistencies regarding to differences in the categorization of expertise in RA as a variable (continuous or categorical). Despite all these limitations, our data represents real-world experience from an unselected cohort of patients from the national database, which is different from randomized controlled trials. Thus, our results could be extrapolated to the general population.

## 7. Conclusions

Higher experience in RA might be linked to worse outcome in PCI via FA in both SA and ACS settings. Presented data suggest that increasing unfamiliarity with the FA is detrimental on clinical outcome. Femoral artery is an important vascular approach and should not be abandoned while learning procedures with RA. Operators on training should be encouraged to develop proficiency in both RA and FA.

## Figures and Tables

**Table 1:** Baseline patient characteristics treated by operators divided into quartiles depending on operator’s utilization of radial access site.

Variable	Quartiles				*p*-Value
	<25%	25–50%	50–75%	>75%	
Male gender	67.6%	68.8%	67.9%	67.7%	0.001
Age (years)	67.0 (±10.7)	67.0 (±10.8)	67.1 (±10.8)	67.2 (±10.9)	0.004
Diabetes mellitus	23.0%	23.2%	24.5%	25.3%	0.001
Previous stroke	3.0%	3.2%	3.4%	3.3%	0.001
Previous MI	29.7%	30.8%	30.9%	33.0%	0.001
Previous CABG	7.8%	6.4%	5.6%	5.6%	0.001
Previous PCI	37.3%	38.1%	38.2%	37.3%	0.001
Smoking	18.1%	18.8%	18.5%	21.7%	0.001
Arterial hypertension	74.1%	71.0%	69.7%	71.4%	0.001
Chronic kidney disease	5.3%	5.3%	4.6%	6.1%	0.001
Chronic obstructive pulmonary disease	2.1%	2.3%	2.4%	2.9%	0.001

Data are presented as percentage or mean and standard deviation. CABG—coronary artery bypass grafting; MI—myocardial infarction; PCI—percutaneous coronary intervention.

**Table 2:** Treatment during percutaneous coronary intervention and angiographic characteristic of patients treated by operators divided into quartiles depending on operator’s utilization of radial access site.

Variable	Quartiles				*p*-Value
	<25%	25–50%	50–75%	>75%	
Acetylsalicylic acid	35.2%	32.8%	27.8%	35.5%	0.001
P2Y_12_ inhibitors					
Clopidogrel	52.6%	34.0%	44.0%	42.7%	0.001
Prasugrel	0.8%	0.6%	0.5%	0.3%	0.001
Ticagrelor	6.9%	5.1%	4.0%	3.6%	0.001
Unfractionated heparin	85.2%	87.1%	83.1%	83.0%	0.001
Bivalirudin	0.4%	0.5%	0.2%	0.2%	0.001
Glycoprotein IIb/IIIa inhibitors	2.8%	3.0%	3.4%	3.2%	0.001
Single-vessel disease	84.0%	85.4%	84.7%	86.1%	0.001
LMCA only	1.2%	1.2%	1.4%	1.4%	0.001
Multi-vessel disease without LMCA	8.6%	9.0%	8.6%	8.5%	0.001
Multi-vessel disease with LMCA	1.9%	2.0%	1.7%	1.5%	0.001

Data are presented as percentage. LMCA—left main coronary artery.

**Table 3:** Clinical outcomes of angiography procedure performed by operators divided into quartiles depending on operator’s utilization of radial access site.

Variable	Quartiles				*p*-Value
	<25% (*n* = 33)	25–50% (*n* = 62)	50–75% (*n* = 143)	>75% (*n* = 449)	
RA during angiography, %	12.87 (±6.43)	38.70 (±7.11)	64.41 (±7.64)	89.72 (±5.86)	0.001
FA during angiography, %	87.13 (±6.43)	61.30 (±7.11)	35.59 (±7.64)	10.28 (±5.86)	0.001
Death during angiography procedure, %	0.21 (±0.24)	0.21 (±0.28)	0.27 (±0.31)	0.18 (±0.18)	0.09
Death during angiography procedures with RA, %	0.05 (±0.19)	0.06 (±0.14)	0.10 (±0.19)	0.10 (±0.13)	0.001
Death during angiography procedures with FA, %	0.24 (±0.28)	0.30 (±0.46)	0.59 (±0.72)	1.05 (±2.09)	0.008
Stroke during angiography %	0.01 (±0.04)	0.02 (±0.04)	0.02 (±0.05)	0.01 (±0.11)	0.09
Stroke during angiography procedures with RA, %	0.02 (±0.12)	0.01 (±0.05)	0.01 (±0.05)	0.01 (±0.08)	0.5
Stroke during angiography procedures with FA, %	0.01 (±0.05)	0.03 (±0.07)	0.03 (±0.11)	0.06 (±0.96)	0.001
Bleeding at the puncture site during angiography, %	0.02 (±0.05)	0.06 (±0.11)	0.07 (±0.19)	0.03 (±0.08)	0.008
Bleeding at the puncture site during angiography procedures with RA, %	0.00 (±0.00)	0.03 (±0.11)	0.04 (±0.13)	0.02 (±0.07)	0.1
Bleeding at the puncture site during angiography procedures with FA, %	0.02 (±0.06)	0.07 (±0.14)	0.13 (±0.38)	0.12 (±0.60)	0.001

Data are presented as a number (percentage) or mean and standard deviation. ACS—acute coronary syndrome; FA—femoral access; RA—Radial access; SA—stable angina.

**Table 4:** Clinical outcomes of percutaneous coronary intervention performed by operators divided into quartiles depending on operator’s utilization of radial access site.

Variable	Quartiles				*p*-Value
	<25% (*n* = 34)	25–50% (*n* = 67)	50–75% (*n* = 112)	>75% (*n* = 326)	
RA during PCI, %	12.16 (±6.10)	38.42 (±7.81)	64.39 (±7.34)	88.05 (±5.75)	0.001
FA during PCI, %	87.84 (±6.10)	61.58 (±7.81)	35.61 (±7.34)	11.95 (±5.75)	0.001
Death during PCI, %	0.24 (±0.26)	0.44 (±0.51)	0.37 (±0.40)	0.28 (±0.27)	0.02
Death during PCI procedures with RA, %	0.07 (±0.30)	0.19 (±0.38)	0.14 (±0.24)	0.16 (±0.21)	0.001
Death during PCI procedures with FA, %	0.26 (±0.29)	0.60 (±0.69)	0.78 (±0.81)	1.41 (±2.11)	0.003
Bleeding at the puncture site during PCI, %	0.13 (±0.35)	0.17 (±0.37)	0.07 (±0.20)	0.10 (±0.22)	0.2
Bleeding at the puncture site during PCI procedures with RA, %	0.05 (±0.22)	0.11 (±0.30)	0.04 (±0.12)	0.06 (±0.17)	0.08
Bleeding at the puncture site during PCI procedures with FA, %	0.14 (±0.37)	0.21 (±0.45)	0.14 (±0.36)	0.43 (±1.09)	0.3

Data are presented as a number (percentage) or mean and standard deviation. ACS—acute coronary syndrome; FA—femoral access; PCI—percutaneous coronary intervention; RA—Radial access; SA—stable angina.

**Table 5:** Clinical outcomes of angiography performed by operators divided into quartiles depending on operator’s utilization of radial access site. Data presented separately for stable angina and acute coronary syndrome.

Variable	Quartiles				*p*-Value
	<25% (*n* = 33)	25–50% (*n* = 62)	50–75% (*n* = 143)	>75% (*n* = 449)	
Death during angiography procedures in ACS group with RA, %	0.05 (±0.29)	0.09 (±0.27)	0.17 (±0.34)	0.17 (±0.23)	0.001
Death during angiography procedures in ACS group with FA, %	0.42 (±0.58)	0.46 (±0.62)	0.86 (±1.16)	1.38 (±2.54)	0.2
Death during angiography procedures in SA group with RA, %	0.05 (±0.28)	0.00 (±0.00)	0.02(±0.12)	0.01 (±0.04)	0.5
Death during angiography procedures in SA group with FA, %	0.03 (±0.11)	0.06 (±0.32)	0.09 (±0.59)	0.04 (±0.45)	0.004
Stroke during angiography procedures in ACS group with RA, %	0.03 (±0.17)	0.00 (±0.04)	0.02 (±0.08)	0.01 (±0.09)	0.4
Stroke during angiography procedures in ACS group with FA, %	0.02 (±0.07)	0.04 (±0.11)	0.04 (±0.18)	0.07 (±0.98)	0.001
Stroke during angiography procedures in SA group with RA, %	0.00 (±0.00)	0.01 (±0.07)	0.01 (±0.06)	0.01 (±0.07)	0.5
Stroke during angiography procedures in SA group with FA, %	0.00 (±0.00)	0.01 (±0.06)	0.00 (±0.04)	0.01 (±0.14)	0.4
Bleeding at the puncture site during angiography procedures in ACS group with RA, %	0.00 (±0.00)	0.05 (±0.16)	0.05 (±0.17)	0.02 (±0.09)	0.3
Bleeding at the puncture site during angiography procedures in ACS group with FA, %	0.04 (±0.12)	0.09 (±0.24)	0.14 (±0.48)	0.13 (±0.65)	0.003
Bleeding at the puncture site during angiography procedures in SA group with RA, %	0.00 (±0.00)	0.00 (±0.00)	0.03 (±0.15)	0.03 (±0.16)	0.03
Bleeding at the puncture site during angiography procedures in SA group with FA, %	0.01 (±0.05)	0.05 (±0.14)	0.12 (±0.47)	0.15 (±1.65)	0.01

ACS—acute coronary syndrome; FA—femoral access; PCI—percutaneous coronary intervention; RA—Radial access; SA—stable angina.

**Table 6:** Clinical outcomes of percutaneous coronary intervention performed by operators divided into quartiles depending on operator’s utilization of radial access site. Data presented separately for stable angina and acute coronary syndrome.

Variable	Quartiles				*p*-Value
	<25% (*n* = 34)	25–50% (*n* = 67)	50–75% (*n* = 112)	>75% (*n* = 326)	
Death during PCI procedures in ACS group with RA, %	0.10 (±0.42)	0.24 (±0.48)	0.18 (±0.30)	0.21 (±0.28)	0.001
Death during PCI procedures in ACS group with FA, %	0.31 (±0.40)	0.68 (±0.73)	0.93 (±1.05)	1.63 (±2.52)	0.01
Death during PCI procedures in SA group with RA, %	0.00 (±0.00)	0.05 (±0.28)	0.02 (±0.15)	0.02 (±0.09)	0.6
Death during PCI procedures in SA group with FA, %	0.10 (±0.29)	0.13 (±0.65)	0.31 (±2.38)	0.04 (±0.44)	0.005
Bleeding at the puncture site during PCI procedures in ACS group with RA, %	0.08 (±0.33)	0.10 (±0.28)	0.05 (±0.18)	0.07 (±0.20)	0.1
Bleeding at the puncture site during PCI procedures in ACS group with FA, %	0.15 (±0.40)	0.22 (±0.47)	0.14 (±0.40)	0.37 (±1.11)	0.09
Bleeding at the puncture site during PCI procedures in SA group with RA, %	0.00 (±0.00)	0.09 (±0.48)	0.02 (±0.12)	0.05 (±0.28)	0.2
Bleeding at the puncture site during PCI procedures in SA group with FA, %	0.11 (±0.36)	0.14 (±0.52)	0.12 (±0.44)	0.65 (±2.94)	0.08

Data are presented as a number (percentage) or mean and standard deviation. ACS—acute coronary syndrome; FA—femoral access; PCI—percutaneous coronary intervention; RA—Radial access; SA—stable angina.

**Table 7:** Comparison of event rates between procedures with radial and femoral access site for stable angina and acute coronary syndrome in a group of operators performing ≤25% of procedures with radial artery.

Variable	Angiography			Percutaneous Coronary Intervention		
	Radial (*n* = 33)	Femoral (*n* = 33)	*p*-Value	Radial (*n* = 34)	Femoral (*n* = 34)	*p*-Value
Death during procedure	0.05 (±0.19)	0.24 (±0.28)	0.001	0.07 (±0.30)	0.26 (±0.29)	0.002
Bleeding at the puncture site during procedure	0.00 (±0.00)	0.02 (±0.06)	0.02	0.05 (±0.22)	0.14 (±0.37)	0.003
Stroke during procedure	0.02 (±0.12)	0.01 (±0.05)	0.6	0	0	-
	Stable angina					
Death during procedure	0.05 (±0.28)	0.03 (±0.11)	0.6	0.00 (±0.00)	0.10 (±0.29)	0.04
Bleeding at the puncture site during procedure	0.00 (±0.00)	0.01 (±0.05)	0.2	0.00 (±0.00)	0.11 (±0.36)	0.04
Stroke during procedure	0	0	-	0	0	-
	Acute coronary syndrome					
Death during procedures	0.05 (±0.29)	0.42 (±0.58)	0.001	0.10 (±0.42)	0.31 (±0.40)	0.001
Stroke during procedure	0.03 (±0.17)	0.02 (±0.07)	0.6	0	0	-
Bleeding at the puncture site during procedure	0.00 (±0.00)	0.04 (±0.12)	0.04	0.08 (±0.33)	0.15 (±0.40)	0.08

Data are presented as a number (percentage) or mean and standard deviation. ACS—acute coronary syndrome; FA—femoral access; PCI—percutaneous coronary intervention; RA—Radial access; SA—stable angina.

**Table 8:** Comparison of event rates between procedures with radial and femoral access site for stable angina and acute coronary syndrome in a group of operators performing 25–50% of procedures with radial artery.

Variable	Angiography			Percutaneous Coronary Intervention		
	Radial (*n* = 62)	Femoral (*n* = 62)	*p*-Value	Radial (*n* = 67)	Femoral (*n* = 67)	*p*-Value
Death during procedure	0.06 (±0.14)	0.30 (±0.46)	0.001	0.19 (±0.38)	0.60 (±0.69)	0.001
Bleeding at the puncture site during procedure	0.03 (±0.11)	0.07 (±0.14)	0.01	0.11 (±0.30)	0.21 (±0.45)	0.004
Stroke during procedure	0.01 (±0.05)	0.03 (±0.07)	0.1	0	0	-
	Stable angina					
Death during procedure	0.00 (±0.00)	0.06 (±0.32)	0.045	0.05 (±0.28)	0.13 (±0.65)	0.2
Bleeding at the puncture site during procedure	0.00 (±0.00)	0.05 (±0.14)	0.007	0.09 (±0.48)	0.14 (±0.52)	0.5
Stroke during procedure	0.01 (±0.07)	0.01 (±0.06)	0.6	0	0	-
	Acute coronary syndrome					
Death during procedures	0.09 (±0.27)	0.46 (±0.62)	0.001	0.24 (±0.48)	0.68 (±0.73)	0.001
Stroke during procedure	0.00 (±0.04)	0.04 (±0.11)	0.01	0	0	-
Bleeding at the puncture site during procedure	0.05 (±0.16)	0.09 (±0.24)	0.5	0.10 (±0.28)	0.22 (±0.47)	0.003

Data are presented as a number (percentage) or mean and standard deviation. ACS—acute coronary syndrome; FA—femoral access; PCI—percutaneous coronary intervention; RA—Radial access; SA—stable angina.

**Table 9:** Comparison of event rates between procedures with radial and femoral access site for stable angina and acute coronary syndrome in a group of operators performing 50–75% of procedures with radial artery.

Variable	Angiography			Percutaneous Coronary Intervention		
	Radial (*n* = 143)	Femoral (*n* = 143)	*p*-Value	Radial (*n* = 112)	Femoral (*n* = 112)	*p*-Value
Death during procedure	0.10 (±0.19)	0.59 (±0.72)	0.001	0.14 (±0.24)	0.78 (±0.81)	0.001
Bleeding at the puncture site during procedure	0.04 (±0.13)	0.13 (±0.38)	0.003	0.04 (±0.12)	0.14 (±0.36)	0.009
Stroke during procedure	0.01 (±0.05)	0.03 (±0.11)	0.6	0	0	-
	Stable angina					
Death during procedure	0.02 (±0.12)	0.09 (±0.59)	0.2	0.02 (±0.15)	0.31 (±2.38)	0.1
Bleeding at the puncture site during procedure	0.03 (±0.15)	0.12 (±0.47)	0.04	0.02 (±0.12)	0.12 (±0.44)	0.053
Stroke during procedure	0.01 (±0.06)	0.00 (±0.04)	0.3	0	0	-
	Acute coronary syndrome					
Death during procedures	0.17 (±0.34)	0.86 (±1.16)	0.001	0.18 (±0.30)	0.93 (±1.05)	0.001
Stroke during procedure	0.02 (±0.08)	0.04 (±0.18)	0.9	0	0	-
Bleeding at the puncture site during procedure	0.05 (±0.17)	0.14 (±0.48)	0.02	0.05 (±0.18)	0.14 (±0.40)	0.056

Data are presented as a number (percentage) or mean and standard deviation. ACS—acute coronary syndrome; FA—femoral access; PCI—percutaneous coronary intervention; RA—Radial access; SA—stable angina.

**Table 10:** Comparison of event rates between procedures with radial and femoral access site for stable angina and acute coronary syndrome in a group of operators performing ≥75% of procedures with radial artery.

Variable	Angiography			Percutaneous Coronary Intervention		
	Radial (*n* = 449)	Femoral (*n* = 449)	*p*-Value	Radial (*n* = 326)	Femoral (*n* = 326)	*p*-Value
Death during procedure	0.10 (±0.13)	1.05 (±2.09)	0.001	0.16 (±0.21)	1.41 (±2.11)	0.001
Bleeding at the puncture site during procedure	0.02 (±0.07)	0.12 (±0.60)	0.5	0.06 (±0.17)	0.43 (±1.09)	0.001
Stroke during procedure	0.01 (±0.08)	0.06 (±0.96)	0.001	0	0	-
	Stable angina					
Death during procedure	0.01 (±0.04)	0.04 (±0.45)	0.08	0.02 (±0.09)	0.04 (±0.44)	0.01
Bleeding at the puncture site during procedure	0.03 (±0.16)	0.15 (±1.65)	0.03	0.05 (±0.28)	0.65 (±2.94)	0.1
Stroke during procedure	0.01 (±0.07)	0.01 (±0.14)	0.001	0	0	-
	Acute coronary syndrome					
Death during procedures	0.17 (±0.23)	1.38 (±2.54)	0.001	0.21 (±0.28)	1.63 (±2.52)	0.001
Stroke during procedure	0.01 (±0.09)	0.07 (±0.98)	0.02	0	0	-
Bleeding at the puncture site during procedure	0.02 (±0.09)	0.13 (±0.65)	0.5	0.07 (±0.20)	0.37 (±1.11)	0.3

Data are presented as a number (percentage) or mean and standard deviation. ACS—acute coronary syndrome; FA—femoral access; PCI—percutaneous coronary intervention; RA—Radial access; SA—stable angina.
